# Molecular characterization of flaviviruses from field-collected mosquitoes in northwestern Italy, 2011–2012

**DOI:** 10.1186/1756-3305-7-395

**Published:** 2014-08-26

**Authors:** Francesca Rizzo, Francesco Cerutti, Marco Ballardini, Andrea Mosca, Nicoletta Vitale, Maria Cristina Radaelli, Rosanna Desiato, Marino Prearo, Alessandra Pautasso, Cristina Casalone, Pierluigi Acutis, Simone Peletto, Maria Lucia Mandola

**Affiliations:** Istituto Zooprofilattico Sperimentale del Piemonte, Liguria e Valle d’Aosta, Via Bologna 148, I-10154 Torino, Italy; Sezione di Imperia, Istituto Zooprofilattico Sperimentale del Piemonte, Liguria e Valle d’Aosta, Via Nizza 4, I-18100 Imperia, Italy; Istituto per le Piante da Legno e l’Ambiente – IPLA, Corso Casale 476, I- 10132 Torino, Italy

**Keywords:** Insect-specific flavivirus, Marisma mosquito virus, Mosquito surveillance, Usutu virus

## Abstract

**Background:**

The genus *Flavivirus* comprises several mosquito-borne species, including the zoonotic pathogens West Nile and Usutu virus, circulating in animals and humans in Italy since 1998. Due to its ecological and geographical features, Piedmont is considered a risk area for flavivirus transmission. Here we report the results of a flavivirus survey (detection and genetic characterization) of mosquitoes collected in Piedmont in 2012 and the genetic characterization of three strains detected in 2011.

**Methods:**

Pools of 1–203 mosquitoes, upon RNA extraction with *TRIzol,* were screened by a PCR assay for a 263 bp fragment of the *Flavivirus* NS5 gene. All positive samples were tested with a specific PCR for the E protein gene of Usutu virus and a generic *Flavivirus* RT-nested-PCR for a larger tract of the NS5 gene before sequencing. Phylogenetic trees were built with both NS5 fragments of representative *Flavivirus* species. DNA extracts of part of the positive pools were tested to detect sequences integrated in the host genome.

**Results:**

Thirty-four mosquito pools resulted positive for flaviviruses, and twenty-five flavivirus sequences underwent phylogenetic analysis for the short NS5 fragment. Among the 19 sequences correlating with the insect-specific flavivirus group, ten samples, retrieved from *Aedes albopictus*, clustered within Aedes flavivirus, while the other nine aggregated in a separate clade composed of strains from various mosquito species (mainly *Aedes vexans*) from Piedmont and the Czech Republic. Six out of these nine also presented a DNA form of the sequence. The remaining sequences belonged to the mosquito-borne group: four, all from *Culex pipiens*, correlated to Italian Usutu virus strains, whereas two, from *Ochlerotatus caspius*, were highly similar to Marisma mosquito virus (MMV).

**Conclusions:**

Our findings confirm the circulation of Usutu virus and of the potentially zoonotic Marisma mosquito virus in Piedmont. This is the first detection of Aedes flavivirus in Piedmont. Finally, further evidence for the integration of *Flavivirus* nucleic acid into the host genome has been shown. These results underline the importance of continuing intense mosquito-based surveillance in Piedmont, supported by a mosquito control program in areas at high risk for human exposure.

## Background

The genus *Flavivirus*, within the family *Flaviviridae*, comprises enveloped, single-stranded RNA viruses [1], most of which are arthropod-borne viruses (arboviruses); it is considered a significant cause of emerging diseases in humans and animals worldwide [2]. Transmitted mainly by mosquitoes and ticks, flaviviruses are able to infect a wide range of hosts, including mammals and birds. The genus includes several major zoonotic pathogens responsible for viral haemorrhagic fevers (i.e., Yellow fever virus and Dengue virus), arboviral encephalitis such as the Tick-borne encephalitis virus, and the Japanese encephalitis group, including West Nile virus (WNV), Usutu virus (USUV), and St. Louis encephalitis virus [3].

Supported by phylogenetic analysis of genome sequences [4, 5], flavivirus species have been grouped according to their antigenic properties and vector associations [6] into mosquito-borne (MBV), tick-borne (TBV), and no-known vector viruses (NKV), as described by Kuno and colleagues [4]. Isolation of novel unclassified flaviviruses from different mosquito species revealed the existence of a novel group diverging from the other known flaviviruses, named insect-specific flavivirus (ISF) [7]. The first described was the Cell fusing agent virus isolated on a mosquito cell culture [8]. To date, the ISF group includes several members, including the Kamiti River virus isolated in Kenya in 1999 [9, 10], Culex flavivirus first isolated from *Culex* mosquitoes in Japan and Indonesia in 2003–2004 [11], and Aedes flavivirus (AeFV) found in *Aedes albopictus* in Japan in 2003–2004 [12]. In Europe, the first ISF isolation was reported in Spain from *Cx.* spp. and *Ochlerotatus caspius* collected in 2006 [13], although genomic sequences had already been detected in *Oc caspius*, *Aedes vexans*, and *Ae. albopictus* in Italy [14, 15], Portugal [16], and Spain [17, 18].

Flavivirus-like sequences integrated in the genome of mosquitoes have been identified in different species of field-collected mosquitoes [14, 19]. The integration of nucleic acid from non-retroviral RNA viruses by eukaryotic cells is a potential evolutionary mechanism that has not been unravelled so far [16, 19].

The MBV group includes several human pathogens (Dengue, Yellow fever, WNV, USUV) able to replicate in vertebrate cells. Recently, novel species like Nounané and Taï forest virus in Cote d’Ivoire [20], Lammi virus in Finland [1], and Marisma mosquito virus (MMV) in Spain [13], detected in field mosquitoes and isolated only on insect cells, were included in the MBV group upon their genetic characterization. As reported in the literature, MMV successfully grew on C6/36 cell lines, whereas its growth on vertebrate lines (VERO and BHK-21) was supported only for the first passage, thus suggesting that MMV is only able to infect insect cells [13].

During the last decades, several arboviral outbreaks in both animals and humans in Europe have mainly been caused by emerging pathogens [21–25], resulting in the establishment of WNV and USUV through a local transmission cycle sustained by vectors and resident bird host populations. Italy has been the scene of several mosquito-borne epidemics: WNV, first detected in 1998 in horses [26], more recently caused neuroinvasive disease in humans in 2008–2013 [27–29]; Chikungunya virus (an *Alphavirus* of the family *Togaviridae*) was identified in *Ae. albopictus* mosquitoes and in humans in 2007 [30, 31]; Usutu virus was associated with the first case of human infection with neurological involvement in 2009 [32, 33].

A recent retrospective analysis detected USUV RNA in tissue samples archived in 1996 during an episode of unusual wild bird mortality in Tuscany (Italy). Partial sequencing confirmed identity with the 2001 Vienna USUV strain and all its descendants, providing evidence for a much earlier introduction of the virus into Europe than previously assumed [34].

The risk factors for flavivirus transmission in northwestern Italy (Piedmont region) include: an abundance of migratory/resident avifauna and mosquito species (e.g., *Aedes* spp*.* and *Culex* spp*.*), vectors competent for arbovirus transmission [35], and geographical features (Alps, rivers, lakes, extensive rice fields). Preliminary evidence for USUV and flavivirus infection in mosquitoes in Piedmont and bordering regions [15, 36] prompted the establishment of a monitoring program in 2011 based on the integrated serological and virological surveillance of reservoir birds, horses and mosquitoes. During the first year of monitoring, WNV and USUV antibodies were detected in horses [37], and flavivirus RNA (including USUV) was found at a very low prevalence in mosquitoes, but no further molecular characterization was performed [38].

Here, we present the results of virological surveillance, in terms of flavivirus presence and prevalence, carried out on mosquitoes in Piedmont during the summer of 2012. In order to gain better insight into the evolutionary relationship of circulating mosquito flavivirus species, molecular characterization and phylogenetic analysis were performed on the strains detected in 2011 and 2012.

## Methods

### Survey area and mosquito collection

**Figure 1:**
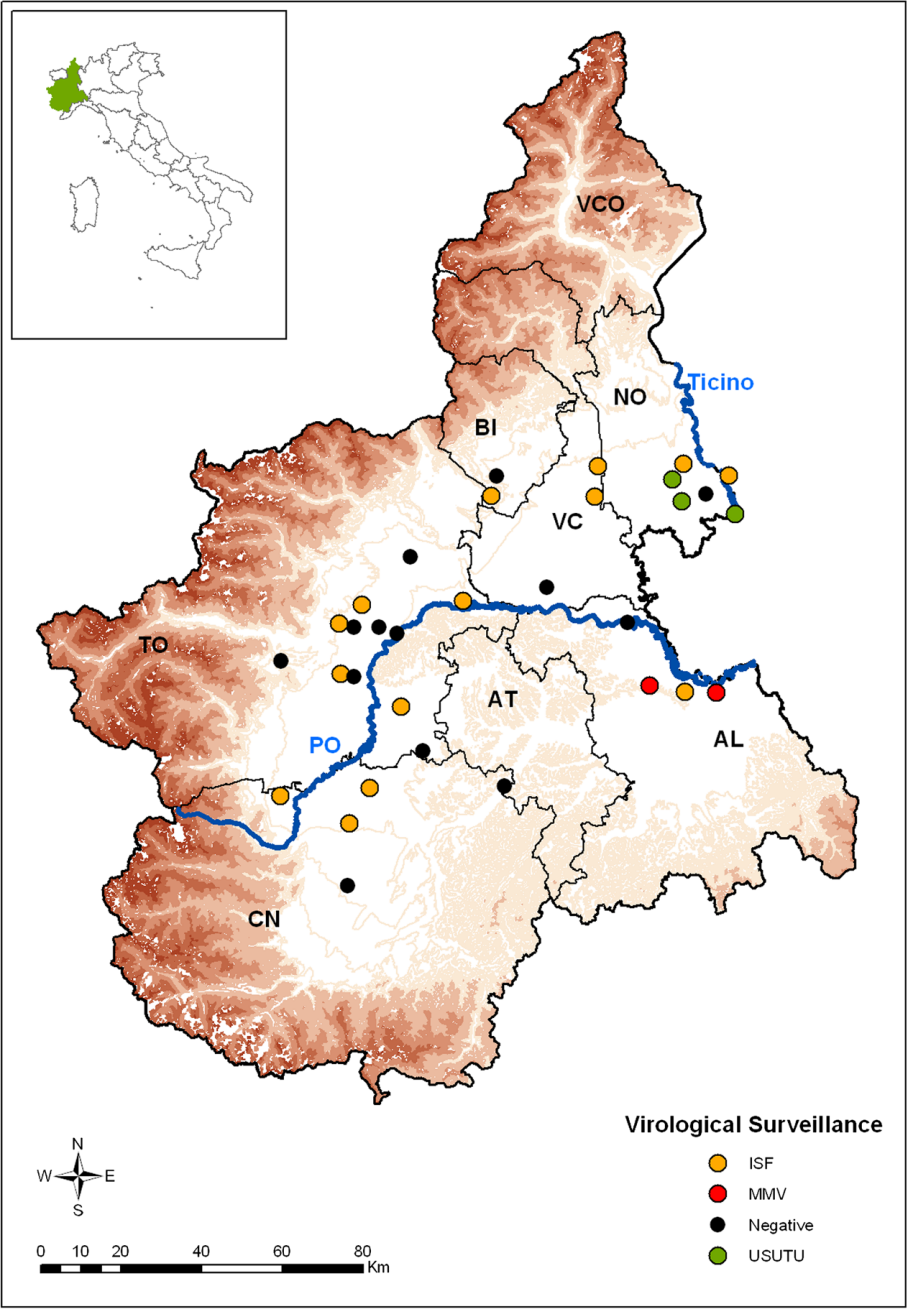
**Map of Piedmont.** The map indicates the trap sites for the virological surveillance. Province borders are defined and indicated as TO: Torino, CN: Cuneo, AT: Asti, AL: Alessandria, VC: Vercelli, NO: Novara, BI: Biella, VCO: Verbano-Cusio-Ossola. The 32 virological traps are colour-coded according to the results of flavivirus detection: black for negative traps, red for MMV-positive traps, yellow for ISF-positive traps, green for USUV-positive traps.

Piedmont*,* in northwestern Italy*,* is divided into eight provinces (total area, 25,401.56 km^2^; population, 4,457,335 inhabitants). It is located at the western end of the Po Plain and is surrounded by the Alps on three sides (Figure 1). It is crossed by the Po river and bounded to the east by the Ticino river; it borders two other regions, Lombardy and Emilia-Romagna, with established and documented flavivirus circulation. Based on previous studies, which reported flavivirus circulation in mosquitoes in late summer [15, 36, 38], the virological surveillance started on August 1st and sampling was performed every two weeks till October 12th, 2012. The number of CO_2_-baited traps selected in 2012 for flavivirus surveillance was increased to 32 (i.e., 17 more than in the previous year) in order to extend coverage of the region. The traps were placed in locations at high risk for the establishment of the vector-host flavivirus transmission cycle, according to risk-based factors such as proximity to wet areas (rice fields, Po and Ticino rivers), airports, farms with previous WNV seropositivity in horses, and an abundance of migratory and resident avifauna.

Species identification was based on morphological characteristics by means of standard classification keys [39, 40]. Mosquitoes were maintained under cold chain conditions to preserve virus viability in the samples, pooled in a maximum of 203 specimens according to date, trap location and species, and stored at -80°C. Each pool was ground in 2 mL of PBS plus two 4.5 mm copper beads in a TissueLyser (Qiagen); the homogenates were clarified by centrifugation, and 200 μL of each supernatant were collected and stored in TRIzol^®^ (Sigma-Aldrich) at -80°C until processing.

### Virus identification

RNA extraction was performed with 600 μL of TRIzol^®^ (Sigma-Aldrich) with a final elution in 50 μL of DNase-RNase-free water. A two-step amplification was set up with the reverse transcription (RT) step performed using the High-capacity cDNA Reverse Transcription kit (Life Technologies), according to the manufacturer’s instructions.

In order to detect both generic flavivirus and specific West Nile virus infection, cDNA was simultaneously amplified with an end-point PCR assay for the *Flavivirus* genus*,* targeting a 263 bp fragment of the NS5 protein gene [41] and with a real-time PCR assay, targeting a conserved 92 bp fragment of the 3’ noncoding region of WNV [42]. The latter was modified from the original one-step protocol by performing the PCR step after cDNA synthesis. Positive and doubtful samples to the *Flavivirus* PCR were further processed for USUV RNA search using an end-point PCR assay targeting a fragment of 425 bp of the E protein gene [43]. To better characterize these strains, all the positive pools underwent a further generic NS5 RT-nested PCR, targeting a 980 bp region of the NS5 protein gene, according to [13], but reducing the annealing time to 1’ in both the first and second reactions. PCR products were visualized by GelGreen Nucleic Acid Gel Stain (Biotium) staining after electrophoresis on 1.5% high resolution agarose gel w/v, and bands of the expected size were excised from the gel and processed for sequencing.

### DNA extraction and amplification

RNA/DNA extraction was performed on 15 pools resulting as ISF after sequencing of the short NS5 amplicon. The NucleoSpin^®^ RNA II kit (Macherey-Nagel) allows parallel purification of genomic DNA in a separate tube with the NucleoSpin^®^ RNA/DNA Buffer Set, according to the manufacturer’s instructions. A volume of 50 μL of each DNA extract was then digested with RNase A (Qiagen), incubated at 56°C for 10’ and purified on silica columns (Qiagen), washed twice with AW1 and AW2 buffers, and eluted in a final volume of 50 μL of RNase-DNase-free water. Each RNase-treated DNA extract was directly amplified with the end-point PCR assay for the *Flavivirus* genus [41] and amplicons were visualized on 1.5% high resolution agarose gel w/v.

### Calculation of infection rates

Minimum infection rate (MIR) and maximum likelihood estimate (MLE) values were calculated for each positive species using Excel add-in PooledInfRate v3.0 [44] and expressed as the number of infected mosquitoes per 1,000 tested.

### Sequencing and phylogenetic analysis

Three sequences (two MMV from *Oc. caspius* and one USUV from *Cx. pipiens*), obtained during the 2011 surveillance program, were included in the phylogenetic analysis. In order to characterize the detected virus, PCR products were sequenced on an automated fluorescence-based ABI PRISM 3130 Genetic Analyzer (Life Technologies). The obtained sequences were hand-edited using Bioedit software v7.0.5; contigs were then generated with Seqman II 5.00 software (DNASTAR). The sequence identity with available GenBank sequences was computed using the R statistical environment v2.15.2 and the APE package v3.0-8 [45, 46] and expressed as proportional nucleotide identity.

Among all the short NS5 obtained sequences, only high-quality sequences of at least 200 bp were considered for phylogenetic reconstruction and submitted to GenBank. Multiple sequence alignment was composed of 90 sequences (25 from the present study and 65 reference sequences available on GenBank) for a total of 214 sites and was performed using MUSCLE software v3.8.31 [47] supported by Seaview v4.4.0 [48].

A multiple alignment on 937 sites of the long NS5 fragment, including 20 new sequences and 34 reference sequences retrieved from GenBank, was built using Clustal Omega v1.1.0 [49].

To improve the reliability of phylogeny reconstruction in both cases, the best molecular substitution model was selected by jModelTest2 [50, 51] from among the three substitution schemes, considering unequal nucleotide frequency (F), gamma distribution of variation across sites with four categories (G), and proportion of invariant sites (I) for a total of 24 tested models, and the best of NNI and SPR for tree search. The best model was selected by the Akaike information criterion (AIC).

The phylogenetic trees were built under the Bayesian framework implemented in MrBayes v3.2.1, using the jModelTest2 output to set the prior nucleotide substitution model [52, 53]. The other parameters were estimated under default priors, with two runs of four chains each running for 50,000,000 generations. To evaluate the run quality, the convergence of the two runs was verified based on the summary of parameter values, discarding the first 20% of samples as burn-in (sump command implemented in MrBayes), and on Tracer analysis of MCMC traces. The estimated sample size of the likelihood parameter for both tree reconstructions was well over 200, the threshold for significance. MrBayes analyses were performed on the Cipres Science Gateway server [54].

## Results

### Mosquito collection

From August 1st to October 12th, 8533 specimens, belonging to nine mosquito species, were collected from the 32 CO_2_-baited traps selected for analysis in the survey area (Figure 1), as summarized in Table 1. Nineteen traps collected positive mosquitoes, with ten traps collecting positive individuals in more than one session (Table 2).

**Table 1:** **Summary of mosquito specimens collected in 2012 and flaviviral infection rates**

Mosquito species	N° individuals	N° pools	N° positive pools	MIR (95% CI)	MLE (95% CI)
*Ae. albopictus*	232	53	14	60.345	65.831
				(29.703-90.986)	(39.92-102.947)
*Ae. vexans*	164	31	12	73.171	80.464
				(33.315-113.027)	(50.157-126.454)
*An. maculipennis*	405	32	0		
*An. plumbeus*	1	1	0		
*Cx. modestus*	252	9	0		
*Cx. pipiens*	5,610	131	4^a^	0.713	0.739
				(0.15-1.412)	(0.241-1.782)
*Cs. subochrea*	1	1	0		
*Oc. caspius*	1,854	53	2^b^	1.079 (0–2.573)	1.132
					(0.203-3.801)
			1^c^	0.539 (0–1.596)	0.545
					(0.032-2.698)
*Oc. geniculatus*	14	4	1	71.429	83.124
				(0–206.333)	(4.89-502.144)
TOT	8.533	315	34		

^a^ *Cx. pipiens* infected by USUV.

^b^ *Oc. caspius* infected by MMV.

^c^ *Oc. caspius* infected by ISF.

**Table 2:** **Flavivirus positive pools**

Flavivirus strain ID	GenBank accession number	Detected virus	Pool size	Collection date	NS5 short PCR	NS5 long PCR	DNA PCR
**Oc941-AL**	KF882512	MMV	**200**	4 Aug	+	+	/
**Oc942-AL**	KF882513	MMV	**186**	4 Aug/AL	+	+	/
**USU/m2080-NO**	KF882514	USUV	**3**	15 Sept/NO	+	+	/
**Aa564-TO**	KF801589	AeFV	**1**	1 Aug/TO	+	+	/
**Av569-VC**	KF801590	ISF (DNA form)	**11**	1 Aug/VC	+	-	+
**Oc599-AL**	KF801593	MMV	**200**	8 Aug/AL	+	+	/
**Aa607-CN**	KF801591	AeFV	**5**	8 Aug/CN	+	+	/
**Aa610-TO**	KF801592	AeFV	**3**	8 Aug/TO	+	+	/
**USU/m688-NO**	KF801594	USUV	**200**	22 Aug/NO	+	+	/
**USU/m689-NO**	KF801595	USUV	**111**	22 Aug/NO	+	+	/
**Oc694-VC**	/	ISF (DNA form?)	**158**	22 Aug/VC	+	-	ND
**USU/m699-NO**	KF801596	USUV	**203**	22 Aug/NO	+	+	/
**Av707-VC**	/	ISF (DNA form)	**10**	22 Aug/VC	+	-	+
**Og709-VC**	/	ISF (DNA form)	**10**	22 Aug/VC	+	-	+
**Av710-VC**	KF801597	ISF (DNA form)	**4**	22 Aug/	+	-	+
**Oc748-AL**	KF801598	MMV	**88**	28 Aug/AL	+	+	/
**Aa756-CN**	KF801599	AeFV	**2**	29 Aug/CN	+	+	/
**USU/m851-NO**	KF801600	USUV	**18**	5 Sept/NO	+	+	/
**Av852-NO**	/	ISF (DNA form)	**1**	5 Sept/NO	+	-	+
**Av1012-VC**	/	ISF (DNA form?)	**4**	19 Sept/VC	+	-	ND
**Av1014-VC**	KF801601	ISF (DNA form)	**6**	19 Sept/VC	+	-	+
**Aa1015-TO**	/	ISF (DNA form?)	**4**	19 Sept/TO	+	-	ND
**Aa1019-TO**	/	AeFV	**7**	19 Sept/TO	+	ND	/
**Av1020-TO**	KF801602	ISF (DNA form)	**1**	19 Sept/TO	+	-	+
**Aa1028-BI**	KF801603	AeFV	**2**	19 Sept/BI	+	+	/
**Av1106-TO**	KF801604	ISF (DNA form)	**3**	26 Sept/TO	+	-	+
**Aa1108-AL**	KF801605	AeFV	**2**	26 Sept/AL	+	+	/
**Aa1109-TO**	KM206278	AeFV	**6**	26 Sept/TO	+	+	/
**Aa1113-CN**	/	AeFV	**10**	26 Sept/CN	+	ND	/
**Av1116-CN**	KF801606	ISF (DNA form)	**1**	26 Sept/CN	+	-	+
**Av1228-VC**	KF801607	ISF (DNA form)	**4**	3 Oct/VC	+	-	+
**Av1232-NO**	KF801608	ISF (DNA form)	**1**	3 Oct/NO	+	-	+
**Aa1243-TO**	KF801609	AeFV	**4**	5 Oct/TO	+	+	/
**Aa1245-BI**	KF801610	AeFV	**4**	5 Oct/BI	+	+	/
**Aa1326-AL**	KF801611	AeFV	**5**	10 Oct/AL	+	+	/
**Aa1332-AL**	KF801612	AeFV	**2**	10 Oct/AL	+	+	/
**Av1337-NO**	KF801613	ISF (DNA form)	**3**	12 Oct/NO	+	-	+

The Flavivirus strain ID code refers to mosquito species (Oc: *Oc. caspius*, Aa: *Ae. albopictus*, Av: *Ae. vexans*, Og: *Oc. geniculatus*, Cp: *Cx. pipiens*), sample no.- province (TO, Torino; CN, Cuneo; NO, Novara; BI, Biella; VC, Vercelli; AL, Alessandria); for USUV ID: virus/organism (mosquito), sample no.-province. ND: not done for lack of material.

### Virus identification

A total of 315 pools underwent molecular investigations; 34 pools, representing five mosquito species, tested positive on PCR screening for the *Flavivirus* genus, targeting the short NS5 fragment: 14 from *Ae. albopictus*, 12 from *Ae. vexans*, 4 from *Cx. pipiens*, 3 from *Oc. caspius*, and 1 from *Oc. geniculatus.* The prevalence of the detected viruses in each positive mosquito species was evaluated according to the minimum infection rate (MIR) and maximum likelihood estimation (MLE), as summarized in Table 1. The 34 deduced NS5 gene sequences, following a Blast search, identified 28 insect-specific flaviviruses (belonging to different species), 4 USUV, and 2 MMV strains (Table 2). No positivity for WNV was found.

Seventeen out of 34 pools resulted positive also to the long NS5 RT-nested PCR, whereas 15 pools were negative. Two samples had insufficient RNA to perform the analysis.

### DNA extraction and amplification

Twelve out of 15 ISF pools, the same ones that resulted negative to the long NS5 assay, underwent DNA extraction with the RNase treatment and subsequent amplification for the short NS5 fragment. Three pools had insufficient material to perform DNA extraction and amplification. All the tested pools resulted positive to the NS5 short PCR (Table 2).

### Sequencing and phylogenetic analysis

The 28 insect-specific flavivirus short sequences were retrieved from mosquito pools belonging to four species (*Ae. vexans*, *Oc. geniculatus, Oc. caspius*, and *Ae. albopictus*). Thirteen sequences were similar to Aedes flavivirus, while 15 showed a high similarity to uncharacterized flavivirus sequences (Blastn search, data not shown). After manual checking to ensure high quality, 19 sequences were selected for phylogeny reconstruction of the *Flavivirus* genus based on the short NS5 sequence. In this phylogenetic tree, the clade of Aedes flavivirus, within which 10 samples from *Ae. albopictus* clustered, was fully supported by the posterior probability of the common ancestral node. The other nine sequences, all retrieved from *Ae. vexans*, aggregated in a significantly separate clade composed of ISF sequences from different mosquito species collected in Piedmont and the Czech Republic (GenBank: JN802279, JN802281-83). Notably, in five out of nine sequences, the repeat of a 4 bp motif (TTAT) at nucleotide position 9178 (referred to Yellow fever reference strain NC_002031) generated a +1 frameshift, leading to a stop codon correspondent to amino acid at position 66 in the sequences. The same stop codon was present in the sequence AeVe 79 (GenBank: JN802283) from the Czech Republic. All these sequences clustered very closely on the phylogenetic tree.

**Figure 2:**
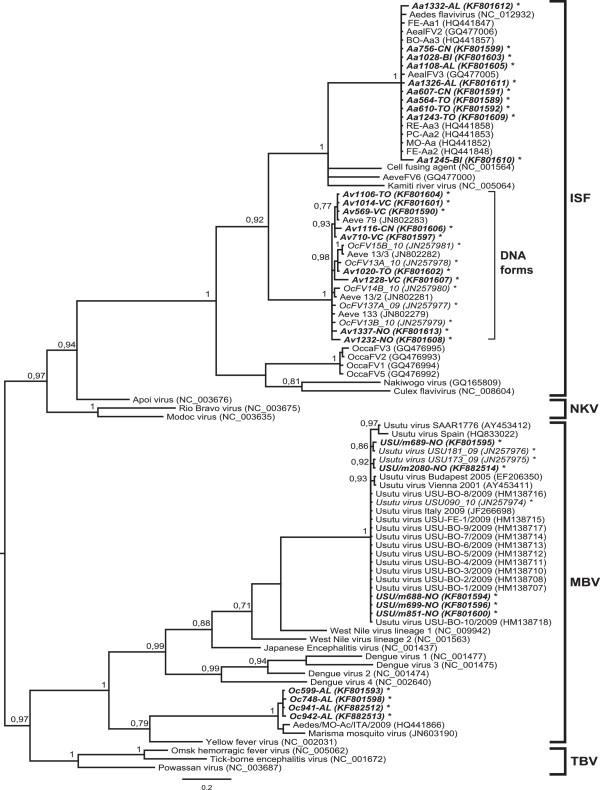
**Phylogenetic tree based on the short NS5 fragment of the Flavivirus genus.** A fragment of 214 bp of the NS5 gene was used to infer the tree. The phylogenetic analysis includes 62 reference sequences from GenBank, 3 sequences from the 2011 surveillance program, and 25 from the present study (19 ISF, 4 USUV, 2 MMV). Unpublished sequences are given in bold, Piedmont sequences in italics. The nucleotide substitution model used as a prior for the MCMC was GTR + G, according to the jModelTest2 results. The tree is mid-point routed; for clarity, only posterior probability of the nodes >0.7 is reported in the figure for statistical support. The scale of branch length is expressed as substitution per site. Taxa are divided into four clusters according to the literature: IS insect-specific; NK no-known vector; MB mosquito-borne; TB tick-borne.

**Figure 3:**
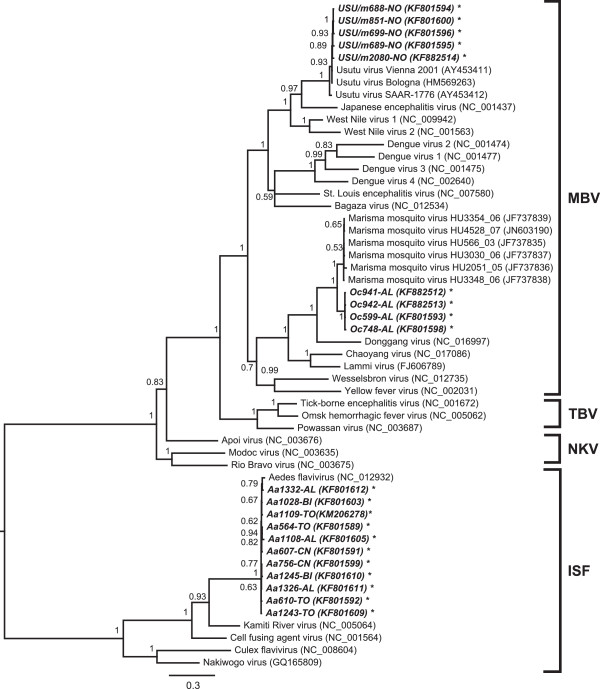
**Phylogenetic tree based on the long NS5 fragment of the Flavivirus genus.** A fragment of 937 bp of the NS5 gene was used to infer the tree. The phylogenetic analysis includes 35 reference sequences from GenBank and 20 from this study. Unpublished sequences are given in bold, Piedmont sequences in italics. The nucleotide substitution model used as a prior for the MCMC was GTR + I + G according to the jModelTest2 results. The tree is mid-point routed, and the scale of branch length is expressed as substitution per site. Taxa are divided into four clusters, according to the literature: IS insect-specific; NK no-known vector; MB mosquito-borne; TB tick-borne.

The five USUV-positive pools were composed of *Cx. pipiens* individuals, all collected in Novara province. In 2012, three pools were sampled on August 22nd (two of which from the same trap in the city of Novara), while in the following session (September 5th) a positive pool was collected in a different location 5 km away from the first ones. The only USUV-positive pool detected in 2011 came from Cameri in Novara province. Positivity was confirmed by USUV-specific PCR assay targeting a portion of the envelope protein (env) gene. The partial env sequences (GenBank: KF801585-88 and KF882515), almost identical to each other, resulted 99% similar to sequences retrieved from *Cx. pipiens* in Piedmont in 2009 (GenBank: JN257983) and 2010 (GenBank: JN257982), as well as to a sequence retrieved from blackbirds in Vienna in 2001 (GenBank: AY453411). However, the similarity decreased to 96% when compared to the SAAR 1776 strain isolated from mosquitoes in South Africa in 1958 (GenBank: AY453412). In the phylogenetic trees of the *Flavivirus* genus, the five USUV strains clustered together with Italian strains previously found in Piedmont and bordering regions (Figures 2 and 3).

Four partial NS5 sequences, two collected in 2011 (GenBank: KF882512-13) and two in 2012 (GenBank: KF801593 and KF801598), all from *Oc. caspius* pools from Alessandria province, showed high similarity to an Italian flavivirus sequence from *Oc. caspius* (Flavivirus Aedes/MO-Ac/ITA/2009, GenBank: HQ441866) and to the Spanish MMV isolate (GenBank: JN603190). Our sequences, 99.5% identical to each other, revealed a 96.5–97% nucleotide identity when compared to HQ441866 and 92–93% to MMV. Phylogenetic analysis of the short fragment confirmed that the four MMV sequences were closely related to the Italian strain and less related to the MMV from Spain (Figure 2), as strongly supported by the posterior probability of the internal node.

To improve genetic characterization and to obtain more information on the relationship of the detected viruses, the long sequences were compared with 34 others from among the most representative flavivirus species in GenBank (Figure 3). The sequences from the Aedes flavivirus species were highly conserved among each other. In the USUV clade, a correlation was observed between collection time and evolutionary trend, although the time factor was not considered in the phylogenetic inference. The Piedmont MMV strain, as in the first tree, lay on a clade separate from the Spanish one, although both were very close to each other.

### Discussion

During the 2012 mosquito survey, we detected 34 positivities, whose nucleotide sequences belong to the *Flavivirus* genus, of which 25 short NS5 sequences were submitted to GenBank and used for a first phylogenetic inference. In addition, we performed genetic characterization of one USUV strain and two MMV strains detected during the 2011 surveillance program. Extending the number of traps placed throughout the whole region allowed us to concentrate our efforts within a narrower time span, demonstrating that by increasing the number of sampling stations per province we were able to obtain a higher detection rate for each province, without drastically increasing the number of captures.

Positivity to the same viral strain in more than one sampling session and in the same mosquito species suggests the establishment of species-specific flavivirus transmission in a particular geographic area. Our phylogenetic analyses, based on both short and long multiple alignments, agree with previous characterization studies on arboviruses from field-collected mosquitoes [4, 15, 36]. These results confirmed the circulation of Aedes flavivirus in Italy, with the first detection in Piedmont. Interestingly, analysis of the other ISF, so far found only in Italy [36] and the Czech Republic, revealed that they are indeed sequences of flavivirus origin integrated into the genomic DNA of at least four mosquito species belonging to two genera, *Aedes* and *Ochlerotatus*. These samples tested positive when their DNA was amplified. Moreover, the long NS5 PCR unsuccessfully amplified their cDNA, supporting the hypothesis for their integration. Furthermore, a non functional stop codon at the 3’ end of the NS5 fragment was observed as the result of repetition of a TTAT motif in five *Ae. vexans* strains from Piedmont and in that from the Czech Republic. This mutation corroborates the integration hypothesis, since the mosquito may not need a functional RNA-dependent RNA polymerase (coded by the NS5 gene); alternatively, it may contribute to immunity to infections by other flavivirus species through the mechanism of RNA interference [55, 56].

In the past, the genus *Ochlerotatus* was classified as a subgenus of the composite genus *Aedes* until it was elevated to the generic rank [57]. The finding of an NS5 sequence integrated in the DNA of these two genera suggests that it might be an ancestral virus that integrated before speciation of these mosquitoes. Nevertheless, as reported by Crochu and colleagues [19], the split between some *Aedes* species occurred much earlier than the estimated origin of the most recent common ancestor of ISF. However, since the two genera are closely related, they may have shared infectious agents in the recent past, and the integration may have occurred in these hosts at different times. Indeed, Cook and colleagues [58] proposed that ISF have experienced multiple introductions with several host-switching events during their evolution. As our sample set was analysed from a surveillance point of view, no further research was performed to investigate this phenomenon.

An interesting result is given by USUV: in both 2011 and 2012 the positive *Cx. pipiens* pools were collected in a small area (10 km^2^) in Novara province, and some pools from the same trap were confirmed positive in both years. The area is in very close proximity to the Ticino river natural park, a wet area of 62.5 km^2^ and an important wintering site for wild waterfowl.

At both the nucleotide and the amino acid levels, the Piedmont USUV sequences have shown only minimal substitutions since 2009, but, based on the long fragment phylogeny, it appears that this virus has slowly evolved over time since its first appearance in Europe. According to recent investigations providing evidence that the first USUV pathogenic outbreak in wild birds dates back to 1996 in Tuscany (Italy), a single introduction event occurred not in Austria in 2001, as was generally assumed, but in Italy as the starting point of the spread of USUV in Europe. This scenario is strengthened by the fact that local herd immunity in Italian wild birds prevented further bird deaths and supported silent spread of the virus in naïve areas. Indeed, large-scale wild bird deaths, as reported for Austria, Switzerland, and Germany [22, 25, 59], were not observed in Italy, despite widespread viral activity [43, 60].

With regard to a previous report [38], detection of MMV was confirmed one year later during the same period (August) and in the same trap, and also detected at a second site 20 km away. Their molecular characterization based on the long NS5 tract, compared to the Spanish strains, showed a genetic distance of less than 10%. On the basis of the molecular classification according to Kuno and colleagues [4], this divergence is low enough to classify the Piedmont strain within the MMV species. Unfortunately, this virus has not yet been well characterized, no further hypotheses can be made, and an appropriate host is lacking.

## Conclusions

Our results confirm the circulation and establishment of Usutu virus in eastern Piedmont due to the presence of the virus within the local *Cx. pipiens* population. The detection of the potentially zoonotic MMV species in the same trap in two consecutive years highlights its presence in a specific geographic area in Alessandria province, but within a widened area, even though a direct connection between the Spanish and Italian strains cannot be made due to lack of data. Furthermore, the detection of integrated DNA of viral origin in the mosquito genome may help to better understand host-pathogen interactions.

Continued intense mosquito-based surveillance in Piedmont, supported by a mosquito control program in areas at high risk for human exposure, is therefore essential. Collection of a large sample of high-quality data is key to determine the genetic relationships among mosquito-borne viruses and evaluate their real zoonotic potential.

## References

[1] Huhtamo E, Putkuri N, Kurkela S, Manni T, Vaheri A, Vapalahti O, Uzcátegui NY (2009). Characterization of a novel flavivirus from mosquitoes in northern europe that is related to mosquito-borne flaviviruses of the tropics. J Virol.

[2] Gubler DJ (2002). The global emergence/resurgence of arboviral diseases as public health problems. Arch Med Res.

[3] Billoir F, de Chesse R, Tolou H, de Micco P, Gould EA, de Lamballerie X (2000). Phylogeny of the genus Flavivirus using complete coding sequences of arthropod-borne viruses and viruses with no known vector. J Gen Virol.

[4] Kuno G, Chang G-JJ, Tsuchiya KR, Karabatsos N, Cropp CB (1998). Phylogeny of the genus Flavivirus. J Virol.

[5] Cook S, Holmes EC (2006). A multigene analysis of the phylogenetic relationships among the flaviviruses (Family: Flaviviridae) and the evolution of vector transmission. Arch Virol.

[6] Calisher CH, Gould EA (2003). Taxonomy of the virus family Flaviviridae. Adv Virus Res.

[7] Cook S, Chung BY-W, Bass D, Moureau G, Tang S, McAlister E, Culverwell CL, Glücksman E, Wang H, Brown TDK, Gould EA, Harbach RE, de Lamballerie X, Firth AE (2013). Novel virus discovery and genome reconstruction from field RNA samples reveals highly divergent viruses in dipteran hosts. PLoS One.

[8] Stollar V, Thomas VL (1975). An agent in the Aedes aegypti cell line (Peleg) which causes fusion of Aedes albopictus cells. Virology.

[9] Sang RC, Gichogo A, Gachoya J, Dunster MD, Ofula V, Hunt a R, Crabtree MB, Miller BR, Dunster LM (2003). Isolation of a new flavivirus related to cell fusing agent virus (CFAV) from field-collected flood-water Aedes mosquitoes sampled from a dambo in central Kenya. Arch Virol.

[10] Crabtree MB, Sang RC, Stollar V, Dunster LM, Miller BR (2003). Genetic and phenotypic characterization of the newly described insect flavivirus, Kamiti River virus. Arch Virol.

[11] Hoshino K, Isawa H, Tsuda Y, Kazuhiko Y, Sasaki T, Yuda M, Takasaki T, Kobayashi M, Sawabe K (2007). Genetic characterization of a new insect flavivirus isolated from Culex pipiens mosquito in Japan. Virology.

[12] Hoshino K, Isawa H, Tsuda Y, Sawabe K, Kobayashi M (2009). Isolation and characterization of a new insect flavivirus from Aedes albopictus and Aedes flavopictus mosquitoes in Japan. Virology.

[13] Vázquez A, Sánchez-Seco M-P, Palacios G, Molero F, Reyes N, Ruiz S, Aranda C, Marqués E, Escosa R, Moreno J, Figuerola J, Tenorio A (2012). Novel flaviviruses detected in different species of mosquitoes in Spain. Vector Borne Zoonotic Dis.

[14] Roiz D, Vázquez A, Seco MPS, Tenorio A, Rizzoli A (2009). Detection of novel insect flavivirus sequences integrated in Aedes albopictus (Diptera: Culicidae) in Northern Italy. Virol J.

[15] Calzolari M, Bonilauri P, Bellini R, Caimi M, Defilippo F, Maioli G, Albieri A, Medici A, Veronesi R, Pilani R, Gelati A, Angelini P, Parco V, Fabbi M, Barbieri I, Lelli D, Lavazza A, Cordioli P, Dottori M (2010). Arboviral Survey of Mosquitoes in Two Northern Italian Regions in 2007 and 2008. Vector Borne Zoonotic Dis.

[16] Parreira R, Cook S, Lopes Â, de Matos AP, de Almeida APG, Piedade J, Esteves A (2012). Genetic characterization of an insect-specific flavivirus isolated from Culex theileri mosquitoes collected in southern Portugal. Virus Res.

[17] Aranda C, Sánchez-Seco MP, Cáceres F, Escosa R, Gálvez JC, Masià M, Marqués E, Ruíz S, Alba A, Busquets N, Vázquez A, Castellà J, Tenorio A (2009). Detection and monitoring of mosquito flaviviruses in Spain between 2001 and 2005. Vector Borne Zoonotic Dis.

[18] Sánchez-Seco M-P, Vázquez A, Collao X, Hernández L, Aranda C, Ruiz S, Escosa R, Marqués E, Bustillo M-A, Molero F, Tenorio A (2010). Surveillance of Arboviruses in Spanish Wetlands: Detection of New Flavi- and Phleboviruses. Vector Borne Zoonotic Dis.

[19] Crochu S, Cook S, Attoui H, Charrel RN, De Chesse R, Belhouchet M, Lemasson J-J, de Micco P, de Lamballerie X (2004). Sequences of flavivirus-related RNA viruses persist in DNA form integrated in the genome of Aedes spp. mosquitoes. J Gen Virol.

[20] Junglen S, Kopp A, Kurth A, Pauli G, Ellerbrok H, Leendertz FH (2009). A new flavivirus and a new vector: characterization of a novel flavivirus isolated from uranotaenia mosquitoes from a tropical rain forest. J Virol.

[21] Hubalek Z, Halouzka J (1999). West Nile fever: a reemerging mosquito-borne viral disease in Europe. Emerg Infect Dis.

[22] Weissenböck H, Kolodziejek J, Url A, Lussy H, Rebel-Bauder B, Nowotny N (2002). Emergence of Usutu virus, an African mosquito-borne flavivirus of the Japanese encephalitis virus group, central Europe. Emerg Infect Dis.

[23] Bakonyi T, Erdélyi K, Ursu K, Ferenczi E, Csörgo T, Lussy H, Chvala S, Bukovsky C, Meister T, Weissenböck H, Nowotny N (2007). Emergence of Usutu virus in Hungary. J Clin Microbiol.

[24] Barzon L, Squarzon L, Cattai M, Franchin E, Pagni S, Cusinato R, Palu G (2009). West Nile virus infection in Veneto region, Italy, 2008–2009. Eurosurveillance.

[25] Becker N, Jöst H, Ziegler U, Eiden M, Höper D, Emmerich P, Fichet-Calvet E, Ehichioya DU, Czajka C, Gabriel M, Hoffmann B, Beer M, Tenner-Racz K, Racz P, Günther S, Wink M, Bosch S, Konrad A, Pfeffer M, Groschup MH, Schmidt-Chanasit J (2012). Epizootic emergence of Usutu virus in wild and captive birds in Germany. PLoS One.

[26] Autorino GL, Battisti A, Deubel V, Ferrari G, Forletta R, Giovannini A, Lelli R, Murri S, Scicluna MT (2002). West Nile virus epidemic in horses, Tuscany region, Italy. Emerg Infect Dis.

[27] Calistri P, Giovannini A, Savini G, Monaco F, Bonfanti L, Ceolin C, Terregino C, Tamba M, Cordioli P, Lelli R (2010). West Nile virus transmission in 2008 in north-eastern Italy. Zoonoses Public Health.

[28] Rizzo C, Salcuni P, Nicoletti L, Ciufolini MG, Russo F, Masala R, Frongia O, Finarelli a C, Gramegna M, Gallo L, Pompa MG, Rezza G, Salmaso S, Declich S (2012). Epidemiological surveillance of West Nile neuroinvasive diseases in Italy, 2008 to 2011. Euro Surveill.

[29] *West Nile bulletin*. http://www.epicentro.iss.it/problemi/westNile/bollettino.asp

[30] Bonilauri P, Bellini R, Calzolari M, Angelini R, Venturi L, Fallacara F, Cordioli P, Angelini P, Venturelli C, Merialdi G, Dottori M (2008). Chikungunya virus in Aedes albopictus, Italy. Emerg Infect Dis.

[31] Rezza G (2009). Chikungunya and West Nile virus outbreaks: what is happening in north-eastern Italy?. Eur J Public Health.

[32] Cavrini F, Gaibani P, Longo G, Pierro A, Rossini G, Bonilauri P, Gerundi G, Di Benedetto F, Pasetto A, Girardis M, Dottori M, Landini MP, Sambri V (2009). Usutu virus infection in a patient who underwent orthotropic liver transplantation, Italy, August-September 2009. Euro Surveill.

[33] Pecorari M, Longo G, Gennari W, Grottola A, Sabbatini A, Tagliazucchi S, Savini G, Monaco F, Simone M, Lelli R, Rumpianesi F (2009). First human case of Usutu virus neuroinvasive infection, Italy, August-September 2009. Euro Surveill.

[34] Weissenböck H, Bakonyi T, Rossi G, Mani P, Nowotny N (2013). Usutu virus, Italy, 1996. Emerg Infect Dis.

[35] Bisanzio D, Giacobini M, Bertolotti L, Mosca A, Balbo L, Kitron U, Vazquez-Prokopec GM (2011). Spatio-temporal patterns of distribution of West Nile virus vectors in eastern Piedmont Region, Italy. Parasit Vectors.

[36] Cerutti F, Giacobini M, Mosca A, Grasso I, Rambozzi L, Rossi L, Bertolotti L (2012). Evidence of mosquito-transmitted flavivirus circulation in piedmont, north-western Italy. Parasit Vectors.

[37] Ministero della Salute e Centro di Referenza Nazionale per lo studio delle Malattie Esotiche Istituto G. Caporale – Teramo (2 Marzo 2012): **Piano Di Sorveglianza Della West Nile Disease (WND) Nelle Areeurbane. Relazione Attività. Gennaio-Dicembre 2011.** http://www.salute.gov.it/imgs/C_17_pubblicazioni_1692_allegato.pdf

[38] Pautasso A, Desiato R, Bertolini S, Vitale N, Radaelli MC, Mancini M, Rizzo F, Mosca A, Calzolari M, Prearo M, Mandola ML, Maurella C, Mignone W, Chiavacci L, Casalone C (2013). Mosquito surveillance in Northwestern Italy to monitor the occurrence of tropical vector-borne diseases. Transbound Emerg Dis.

[39] Severini F, Toma L, Luca M d, Romi R (2009). Identification of the adult stages of the Italian mosquitoes (Diptera, Culicidae). Fragm Entomol.

[40] Stojanovich CJ, Scott HG (1997). Mosquitoes of Italy: Mosquitoes of the Italian Biogeographic Area Which Includes the Republic of Malta, the French Island of Corsica and All of Italy except the Far-Northern Provinces.

[41] Scaramozzino N, Crance JM, Jouan A, DeBriel DA, Stoll F, Garin D (2001). Comparison of flavivirus universal primer pairs and development of a rapid, highly sensitive heminested reverse transcription-PCR assay for detection of flaviviruses targeted to a conserved region of the NS5 gene sequences. J Clin Microbiol.

[42] Tang Y, Anne Hapip C, Liu B, Fang CT (2006). Highly sensitive TaqMan RT-PCR assay for detection and quantification of both lineages of West Nile virus RNA. J Clin Virol.

[43] Manarolla G, Bakonyi T, Gallazzi D, Crosta L, Weissenböck H, Dorrestein G, Nowotny N (2010). Usutu virus in wild birds in northern Italy. Vet Microbiol.

[44] Biggerstaff B (2005). PooledInfRate software. Vector Borne Zoonotic Dis.

[45] Paradis E (2004). APE: analyses of phylogenetics and evolution in R language. Bioinformatics.

[46] R Development Core Team (2008). R: A Language and Environment for Statistical Computing.

[47] Edgar RC (2004). MUSCLE: Multiple sequence alignment with high accuracy and high throughput. Nucleic Acids Res.

[48] Gouy M, Guindon S, Gascuel O (2010). SeaView version 4: A multiplatform graphical user interface for sequence alignment and phylogenetic tree building. Mol Biol Evol.

[49] Sievers F, Wilm A, Dineen D, Gibson TJ, Karplus K, Li W, Lopez R, McWilliam H, Remmert M, Söding J, Thompson JD, Higgins DG (2011). Fast, scalable generation of high-quality protein multiple sequence alignments using Clustal Omega. Mol Syst Biol.

[50] Guindon S, Gascuel O (2003). A simple, fast, and accurate algorithm to estimate large phylogenies by maximum likelihood. Syst Biol.

[51] Darriba D, Taboada GL, Doallo R, Posada D (2012). jModelTest 2: more models, new heuristics and parallel computing. Nat Methods.

[52] Huelsenbeck JP, Ronquist F (2001). MRBAYES: Bayesian inference of phylogenetic trees. Bioinformatics.

[53] Ronquist F, Huelsenbeck JP (2003). MrBayes 3: Bayesian phylogenetic inference under mixed models. Bioinformatics.

[54] Miller MA, Pfeiffer W, Schwartz T (2010). Creating the CIPRES Science Gateway for Inference of Large Phylogenetic Trees. Proceedings of the Gateway Computing Environments Workshop: 14 November 2010; New Orleans.

[55] Ding S-W, Voinnet O (2007). Antiviral immunity directed by small RNAs. Cell.

[56] Campbell CL, Keene KM, Brackney DE, Olson KE, Blair CD, Wilusz J, Foy BD (2008). Aedes aegypti uses RNA interference in defense against Sindbis virus infection. BMC Microbiol.

[57] Reinert JE (2000). New classification for the composite genus Aedes (Diptera: Culicidae: Aedini), elevation of subgenus Ochlerotatus to generic rank, reclassification of the other subgenera, and notes on certain subgenera and species. J Am Mosq Control Assoc.

[58] Cook S, Moureau G, Kitchen A, Gould EA, de Lamballerie X, Holmes EC, Harbach RE (2012). Molecular evolution of the insect-specific flaviviruses. J Gen Virol.

[59] Steinmetz HW, Bakonyi T, Weissenböck H, Hatt J-M, Eulenberger U, Robert N, Hoop R, Nowotny N (2011). Emergence and establishment of Usutu virus infection in wild and captive avian species in and around Zurich, Switzerland–genomic and pathologic comparison to other central European outbreaks. Vet Microbiol.

[60] Savini G, Monaco F, Terregino C, Di Gennaro A, Bano L, Pinoni C, De Nardi R, Bonilauri P, Pecorari M, Di Gialleonardo L, Bonfanti L, Polci A, Calistri P, Lelli R (2011). Usutu virus in ITALY: an emergence or a silent infection?. Vet Microbiol.

